# Homoserine and quorum-sensing acyl homoserine lactones as alternative sources of threonine: a potential role for homoserine kinase in insect-stage Trypanosoma brucei

**DOI:** 10.1111/mmi.12853

**Published:** 2014-11-25

**Authors:** Han B Ong, Wai S Lee, Stephen Patterson, Susan Wyllie, Alan H Fairlamb

**Affiliations:** Division of Biological Chemistry & Drug Discovery, College of Life Sciences, University of DundeeDundee, DD1 5EH, UK

## Abstract

*De novo* synthesis of threonine from aspartate occurs via the β-aspartyl phosphate pathway in plants, bacteria and fungi. However, the *Trypanosoma brucei* genome encodes only the last two steps in this pathway: homoserine kinase (HSK) and threonine synthase. Here, we investigated the possible roles for this incomplete pathway through biochemical, genetic and nutritional studies. Purified recombinant *Tb*HSK specifically phosphorylates L-homoserine and displays kinetic properties similar to other HSKs. HSK null mutants generated in bloodstream forms displayed no growth phenotype *in vitro* or loss of virulence *in vivo*. However, following transformation into procyclic forms, homoserine, homoserine lactone and certain acyl homoserine lactones (AHLs) were found to substitute for threonine in growth media for wild-type procyclics, but not HSK null mutants. The tsetse fly is considered to be an unlikely source of these nutrients as it feeds exclusively on mammalian blood. Bioinformatic studies predict that tsetse endosymbionts possess part (up to homoserine in *Wigglesworthia glossinidia*) or all of the β-aspartyl phosphate pathway (*Sodalis glossinidius*). In addition *S. glossinidius* is known to produce 3-oxohexanoylhomoserine lactone which also supports trypanosome growth. We propose that *T. brucei* has retained HSK and threonine synthase in order to salvage these nutrients when threonine availability is limiting.

## Introduction

Human African trypanosomiasis (African sleeping sickness), a disease caused by two subspecies of the protozoan parasite *Trypanosoma brucei* (*T. b. gambiense* and *T. b. rhodesiense*), is estimated to kill ∼ 10 000 people in sub-Saharan Africa every year (Aksoy, 2011). A third subspecies, *T. b. brucei*, which is non-pathogenic to humans, but causes the economically important cattle disease nagana, is widely used as a model organism for the human disease (Sokolova *et al*., 2010). *T. brucei* infection is transmitted between mammalian hosts via the bite of an infected tsetse fly (*Glossina spp.*), an obligate blood feeder. These parasites undergo marked biological and biochemical changes during their life cycle, alternating predominantly between the bloodstream and procyclic trypomastigote forms in the mammalian bloodstream and tsetse mid-gut respectively (Jones *et al*., 2014).

Current drugs (suramin, pentamidine, melarsoprol and nifurtimox-eflornithine combination therapy) used to treat African sleeping sickness are far from ideal in terms of efficacy, safety and cost (Fairlamb, 2003; Stuart *et al*., 2008). Programmes coordinated by the Drugs for Neglected Diseases initiative (DNDi) have identified two promising candidates (the nitro-drug fexinidazole and the oxaborole SCYX-7158), both of which are currently in clinical development (Barrett, 2010; Nare *et al*., 2010; Maser *et al*., 2012). However, given the high attrition rate in drug discovery, additional potential druggable targets or pathways are required.

**Figure 1:**
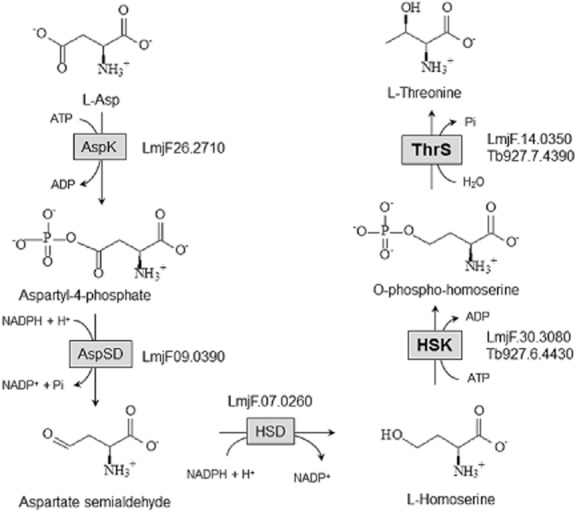
*De novo* threonine biosynthesis pathway. Aspartate is sequentially converted to homoserine via a series of enzymatic reactions involving aspartokinase (AspK), aspartate semialdehyde dehydrogenase (AspSD) and homoserine dehydrogenase (HSD). Homoserine is phosphorylated by HSK to form *O*-phospho-homoserine, a substrate for threonine synthase (ThrS) to produce threonine. Candidate genes for each of these metabolic enzymes are shown for *Trypanosoma brucei* and *Leishmania major*.

One such pathway is the β-aspartyl phosphate pathway found in plants, fungi and bacteria, where aspartate is the precursor for the synthesis of lysine, threonine, methionine and isoleucine (Azevedo *et al*., 2006). This pathway is absent in mammals, and thus these essential amino acids have to be obtained from the diet. The *de novo* biosynthesis of threonine from aspartate involves the key intermediate homoserine (Fig. 1). Homoserine is produced from the sequential phosphorylation of aspartate by aspartokinase (EC 2.7.2.4), followed by the reduction of aspartyl-4-phosphate and aspartate semialdehyde intermediates by aspartate semialdehyde dehydrogenase (EC 1.2.1.11) and homoserine dehydrogenase (EC 1.1.1.3) respectively. Homoserine kinase (HSK, EC 2.7.1.39) then converts homoserine to *O*-phospho-homoserine, which is subsequently metabolised to threonine by threonine synthase (ThrS, EC 4.2.3.1). HSKs are part of the GHMP kinase superfamily that also includes galactokinases, mevalonate kinases and phosphomevalonate kinases. In *Candida albicans*, HSK mutants are hypersensitive to the toxic effects of homoserine and show attenuated virulence in mice (Kingsbury and McCusker, 2010a, b). In the case of another fungal pathogen, *Cryptococcus neoformans*, the threonine biosynthetic pathway is essential (Kingsbury and McCusker, 2008). Thus, HSK is an attractive potential target for drug discovery of novel antifungal compounds (De Pascale *et al*., 2011).

Threonine metabolism is particularly important in African trypanosomes because bloodstream forms preferentially use this amino acid as the major source of acetyl coenzyme A for lipid biosynthesis (Cross *et al*., 1975; Gilbert *et al*., 1983). Although they can salvage threonine from the medium (Voorheis, 1977), it is not known if these parasites can also synthesise it *de novo*. A ^13^C-tracer study demonstrated that aspartate can be efficiently converted to threonine in the related trypanosomatid, *Leishmania mexicana*, via the β-aspartyl phosphate pathway (Saunders *et al*., 2011). Candidate genes for the pathway have been proposed (Fig. 1), including aspartokinase, the first enzyme in the pathway, but none of these have been characterised in trypanosomatids. In contrast, only HSK (Tb927.6.4430) and ThrS (Tb927.7.4390) have been identified in the *T. brucei* genome, and our bioinformatic studies failed to identify any credible candidates for the conversion of aspartate to homoserine.

In the current study, we have used a combination of biochemical and genetic techniques to address a number of questions: does Tb927.6.4430 encode a *bona fide* HSK; is it essential and thus a drug target; where is homoserine derived from; and why would this parasite retain only part of the β-aspartyl phosphate pathway? We provide evidence to suggest that HSK may be important for growth of the insect-stage of the life cycle in which bacterial quorum-sensing molecules produced by a tsetse fly endosymbiont may provide a source of homoserine for threonine biosynthesis.

## Results

### Cloning and sequencing of *Tb*HSK

**Figure 2:**
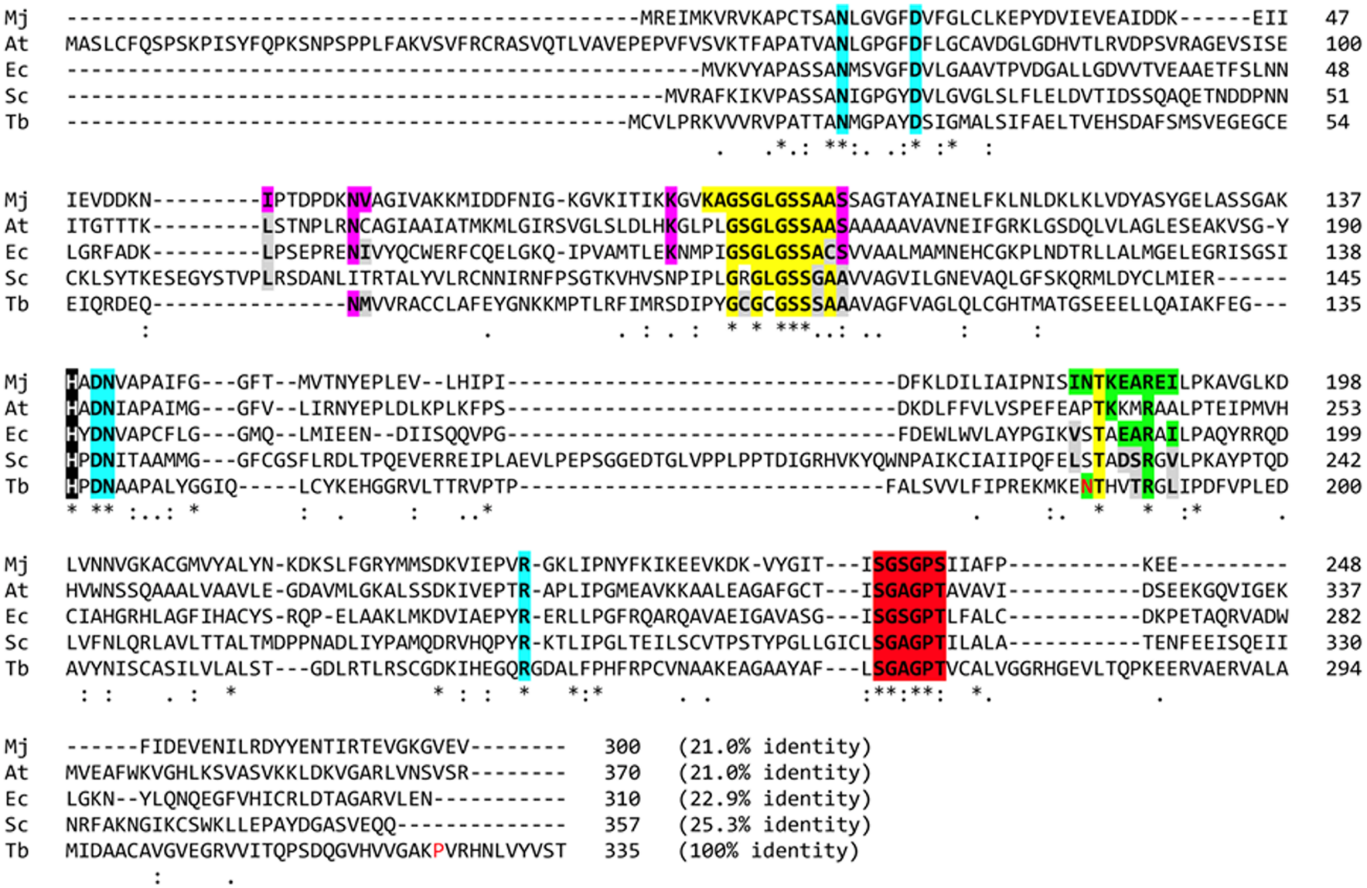
Multiple sequence alignment of HSK from representative species using CLUSTALW. Species abbreviations and NCBI accession numbers: Mj, *Methanococcus jannaschii* (Q58504); At, *Arabidopsis thaliana* (AAD33097); Ec, *Escherichia coli* (YP_488309); Sc, *Saccharomyces cerevisiae* (NP_011890); Tb, *Trypanosoma brucei* (XP_845560). See text for details on highlighted residues.

An alignment of HSK sequences from the *T. brucei* genome strain 927 with representatives from other species is presented in Fig. 2. Key residues identified from structural studies on the *Methanococcus jannaschii* enzyme that are involved in substrate recognition are highlighted (Zhou *et al*., 2000; Krishna *et al*., 2001). Although the sequence identity between *T. brucei* and *M. jannaschii* is low (21%), all five amino acid side chains interacting with homoserine are strictly conserved (highlighted in blue). Significant conservation of the phosphate-binding loop interacting with adenosine triphosphate (ATP) is also evident (yellow), as are two residues in a helix (residues 181–189 in *M. jannaschii*) that undergo pronounced conformational changes upon binding of homoserine, shielding the HSK ternary complex from solvent (green). Another highly conserved loop (residues 259–264 in *M. jannaschii*) is thought to play a role in stabilising the phosphate binding loop (red). A histidine implicated in catalysis is also strictly conserved (white on black).

Sequencing of polymerase chain reaction (PCR) products from our laboratory strain *T. brucei* S427 revealed two sequences containing single nucleotide polymorphisms (SNPs) that resulted in amino acids differences compared with the genome sequence of Tb927.6.4430. A total of 12 clones from three independent PCRs were sequenced and both sequences were found in equal distribution, indicating that there is allelic variation in HSK of diploid *T. brucei* S427. In sequence 1, asparagine^184^ is replaced by serine, while proline^325^ is replaced by serine in sequence 2 (red residues in Fig. 2).

### Kinetic characterisation of *Tb*HSK

**Figure 3:**
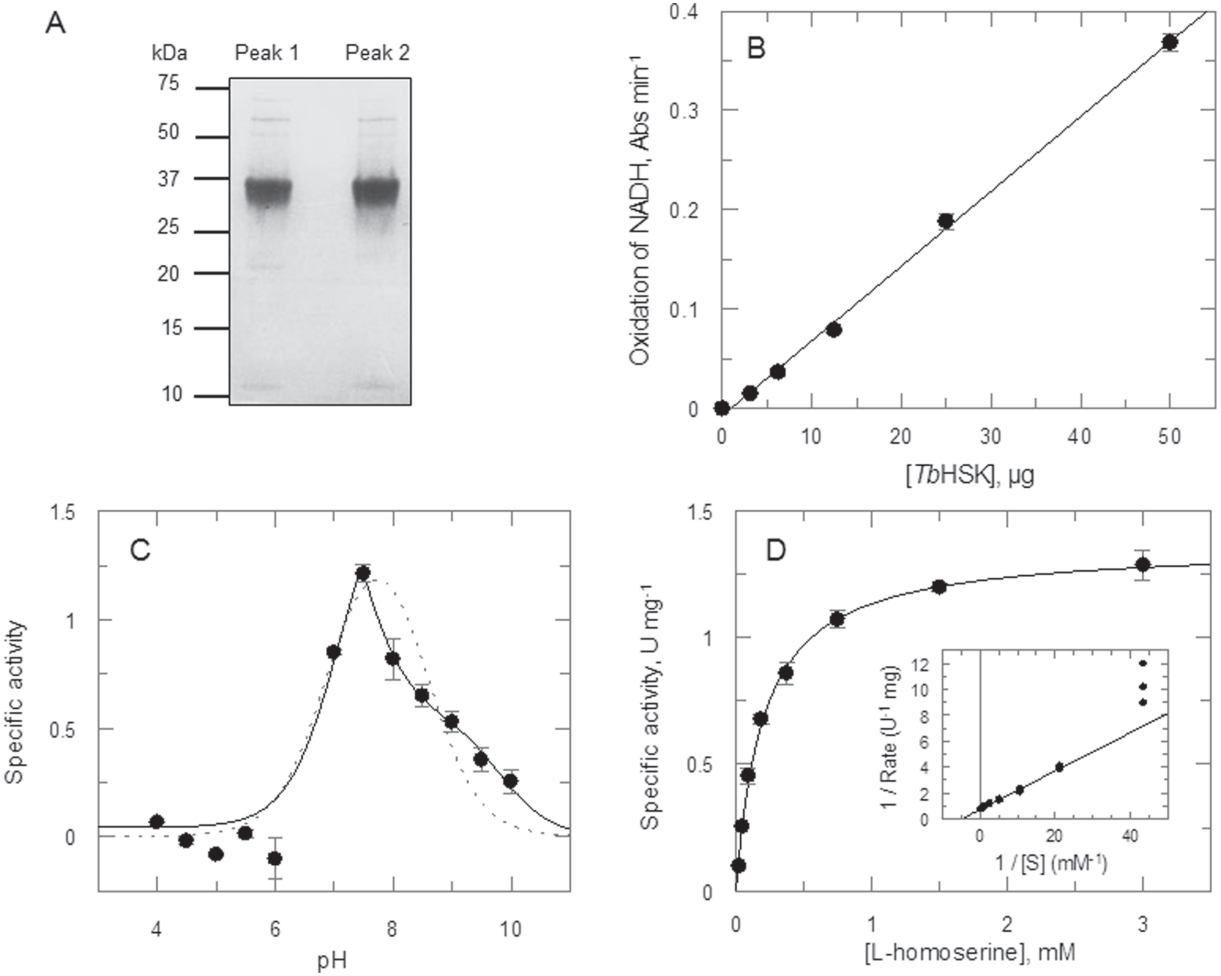
Kinetic characterisation of *Tb*HSK. A. Purity of SDS-PAGE of recombinant *Tb*HSK peaks eluted from the nickel affinity column. B. Rate of the NADH oxidation over a range of *Tb*HSK concentrations. C. pH optimum of *Tb*HSK for L-homoserine determined using constant ionic-strength buffers. D. *K_m_*^app^ of *Tb*HSK for L-homoserine under quasi-physiological conditions.

To confirm that *T. brucei* does indeed encode a *bona fide* HSK, the gene from sequence 1 was expressed with an N-terminal hexa-his tag in *Escherichia coli* and the recombinant protein purified using nickel affinity chromatography. The protein eluted as two separate peaks with > 95% purity as judged by sodium dodecyl sulfate – polyacrylamide gel electrophoresis (SDS-PAGE) (Fig. 3A). The sequence identity of the two protein peaks was verified by tryptic mass fingerprinting with 79% sequence coverage (Proteomic and Mass Spectrometry facility, University of Dundee). The minor contaminating bands were identified as *Escherichia coli* proteins, including chaperonins Hsp70 and GroEL; no *E. coli* HSK was present in an unrelated recombinant protein purified in a similar fashion, so the pooled fractions were deemed suitable for kinetic studies without further purification. Activity was assayed spectrophotometrically at 340 nm by coupling the formation of adenosine diphosphate (ADP) to the oxidation of reduced nicotinamide-adenine dincucleotide (NADH) using phosphoenolpyruvate, pyruvate kinase and lactate dehydrogenase. Under these conditions, *Tb*HSK was found to phosphorylate L-homoserine, with the rate of NADH oxidation linear and directly proportional to the amount of enzyme added (2 to 50 μg ml^−1^, Fig. 3B), yielding a specific activity of 1.2 U mg^−1^. The enzyme showed no activity with other substrates of the GHMP kinase superfamily (galactose, mevalonate and mevalonate phosphate) or with D-homoserine. *Tb*HSK was also unable to phosphorylate other L-amino acids including aspartate, isoleucine, methionine, serine, threonine and valine. Activity was not inhibited by these amino acids at concentrations up to 1 mM. Collectively, these studies confirmed that *Tb*HSK is a *bona fide* homoserine kinase and like other homoserine kinases, is specific for L-homoserine.

*Tb*HSK exhibited a narrow pH profile with optimal activity at pH 7.5 for L-homoserine (Fig. 3C). The results did not fit to a classical bell-shaped pH optimum curve (dotted line), with two inflections above pH7.5. Control experiments showed that the enzyme is stable at the extremes of pH, and thus loss of activity was not due to enzyme inactivation. The double inflections may be due to ionisation of the substrate homoserine (pK_b_ 9.28) together with an amino acid residue in HSK, but this was not investigated further. Nonetheless, the pH optimum profile of *Tb*HSK is in keeping with the reported *T. brucei* intracellular pH of 7.4 (Scott *et al*., 1995). Subsequent characterisation of the enzyme was therefore carried out at this pH. Under these quasi-physiological conditions, *Tb*HSK was found to obey simple Michaelis–Menten kinetics when L-homoserine concentration was varied in presence of a fixed concentration of ATP (3 mM) and enzyme (0.7 μM), yielding a *K_m_*^app^ of 202 ± 10 μM and *k*_cat_ of 0.8 s^−1^ (Fig. 3D). The catalytic efficiency (*k*_cat_/*K*_m_ = 4 × 10^3^ M^−1^ s^−1^) is in good agreement with homoserine kinase from *Arabidopsis thaliana* (2 × 10^3^ M^−1^ s^−1^) (Lee and Leustek, 1999), but lower than *E. coli* (1.3 × 10^5^ M^−1^ s^−1^) (Burr *et al*., 1976) and *Schizosaccharomyces pombe* (2.4 × 10^4^ M^−1^ s^−1^) (De Pascale *et al*., 2011).

### Generation of *HSK* null mutants

**Figure 4:**
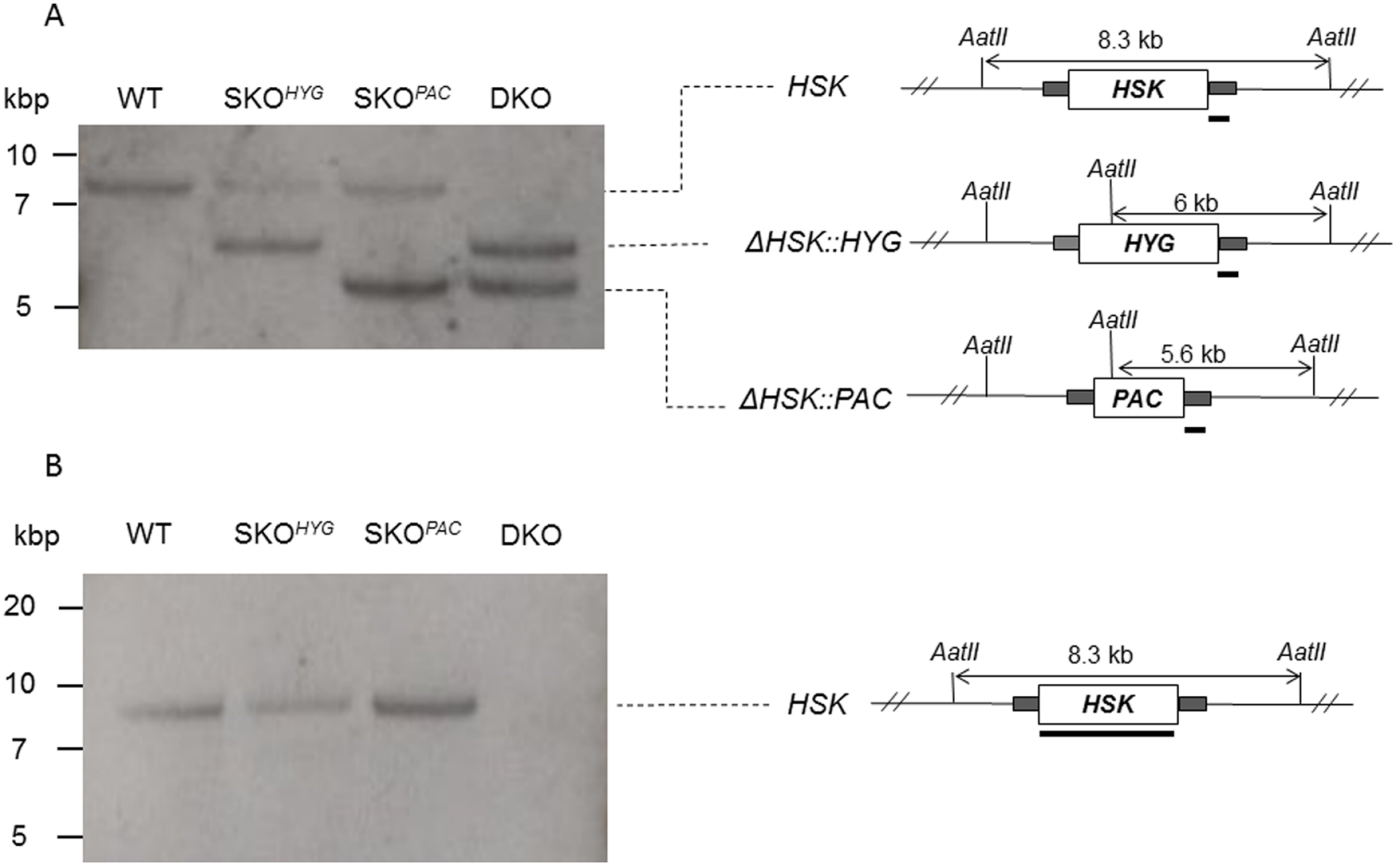
Genotypic analysis of WT, SKO and DKO cell lines. Southern blot analysis of AatII-digested genomic DNA (∼5 μg) from diploid wild-type trypanosomes (WT), Δ*HSK*::*HYG* (SKO^HYG^); Δ*HSK*::*PAC* (SKO^PAC^); and Δ*HSK*::*HYG*/Δ*HSK*::*PAC* (DKO) using a probe against the 3′-UTR of *HSK* (panel A) or the open reading frame of HSK (panel B). A schematic representation of the *HSK* locus and its gene replacements is shown to the right of the blot.

Classical sequential gene replacement was used to investigate whether this enzyme has a role in the growth or survival in bloodstream form *T. brucei*. First, single knockout lines resistant to puromycin (SKO^PAC^) or hygromycin (SKO^HYG^) were generated by transfection with the appropriate gene replacement construct followed by drug selection. After cloning, insertion at the *HSK* locus was confirmed by Southern blot analysis using a probe that hybridises with the 3′-UTR (Fig. 4A). Using the same methodology, a line resistant to both puromycin and hygromycin was obtained following transfection of a cloned SKO^PAC^ line with a *HYG* construct (Fig. 4A). Southern blot analysis using the *HSK* ORF as a probe confirmed that a double knockout null mutant (Δ*HSK*::*PAC*/Δ*HSK*::*HYG*, referred hereafter as DKO) had been obtained (Fig. 4B). Having established that the DKO lacked HSK, cells were cultured in the absence of hygromycin/puromycin for subsequent experiments. In the absence of drug selection, there was no difference in doubling times (both 6.3 h) between DKO and wild-type (WT) cells grown in HMI9T medium.

### Growth analyses of bloodstream *T. brucei*

**Figure 5:**
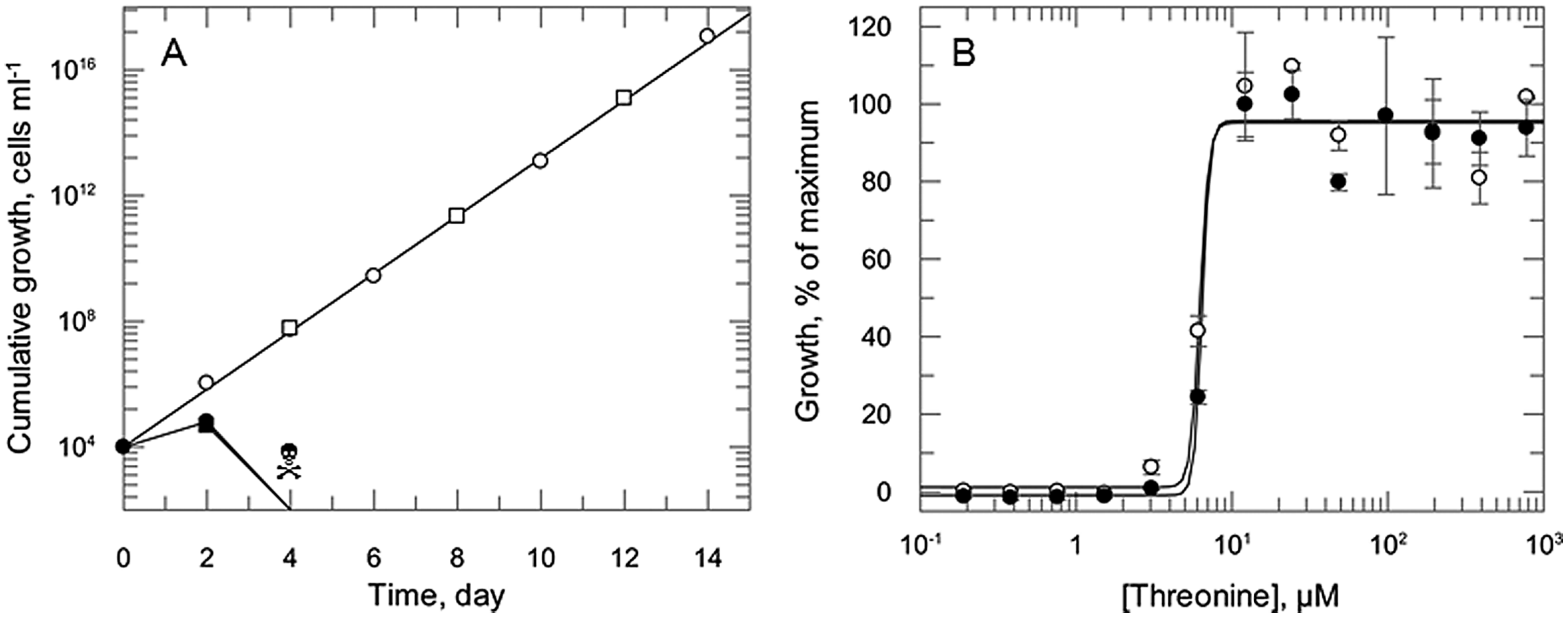
Growth analyses of bloodstream WT and DKO cells *in vitro*. A. WT (circles) and DKO (squares) cells cultured in TBM^TD^ with (open symbols) or without (closed symbols) 800 μM threonine. No viable cells visible after 4 days (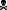). B. Growth of WT (open circles) and DKO (closed circles) cells in varying concentrations of L-threonine.

The ease of generating an *HSK* null mutant agrees with the previous genome-wide study using RNAi (Alsford *et al*., 2011) that HSK is non-essential for parasite survival when grown in rich medium *in vitro*. However, as previously discussed (Ong *et al*., 2013), components of HMI9T may serve as a bypass for loss of *de novo* biosynthesis, making this medium unsuitable for studying more subtle metabolic requirements. In particular, the high concentration of threonine in HMI9T (800 μM) would negate any requirement for *de novo* threonine biosynthesis. With this in mind, parasites were sub-cultured into a threonine-free medium (TBM^TD^) to determine if cells required threonine supplementation for growth. WT cells continued to grow robustly in TBM^TD^ supplemented with 800 μM threonine with a small, but significant (*P* < 0.0001), decrease in doubling time (7.86 ± 0.09 h) compared with cells cultured in HMI9T (6.68 ± 0.04 h). WT cells were unable to grow at all in the absence of threonine with cells perishing by day 4 following subculture (Fig. 5A). DKO parasites grew exactly like WT cells, with the same doubling time and also perished by day 4 without threonine supplementation (Fig. 5A). There was no difference in threonine requirement between these cells (Fig. 5B), with threonine concentrations required for 50% of maximal growth (GC_50_) of 6.2 ± 0.9 and 6.4 ± 1.3 μM for WT and DKO cells respectively. Because the medium contains 200 μM aspartate, these findings suggest that bloodstream *T. brucei* is either unable to synthesise threonine from aspartate or that the rate of *de novo* synthesis pathway is insufficient to meet threonine requirements. In *A. thaliana*, threonine biosynthesis is limited by the rate of homoserine formation from aspartate (Lee *et al*., 2005). To examine whether this was also the case for bloodstream *T. brucei*, cultures in TBM^TD^ were supplemented with varying concentrations of L-homoserine. Unexpectedly, neither WT nor DKO cells were able to utilise L-homoserine for growth, over a wide range of concentrations (0.4 μM to 10 mM). These results indicate that bloodstream form *T. brucei* is incapable of salvaging/utilising homoserine sufficiently to meet the metabolic demand for threonine.

To assess whether HSK is required for virulence, as in the case of *C. albicans* and *Saccharomyces cerevisiae* (Kingsbury and McCusker, 2010a), the infectivity of DKO cells was determined in our animal model of infection. DKO parasites were as infectious as WT cells, with all animals reaching terminal parasitaemia by day 4 of the study. This indicates that the threonine concentration in mouse blood is sufficient to support parasite growth, in agreement with a previous study (Mazet *et al*., 2013). HSK is therefore a non-essential enzyme in bloodstream *T. brucei* both *in vitro* and *in vivo*.

### Procyclic form *T. brucei* can utilise homoserine and homoserine lactones for growth

**Figure 6:**
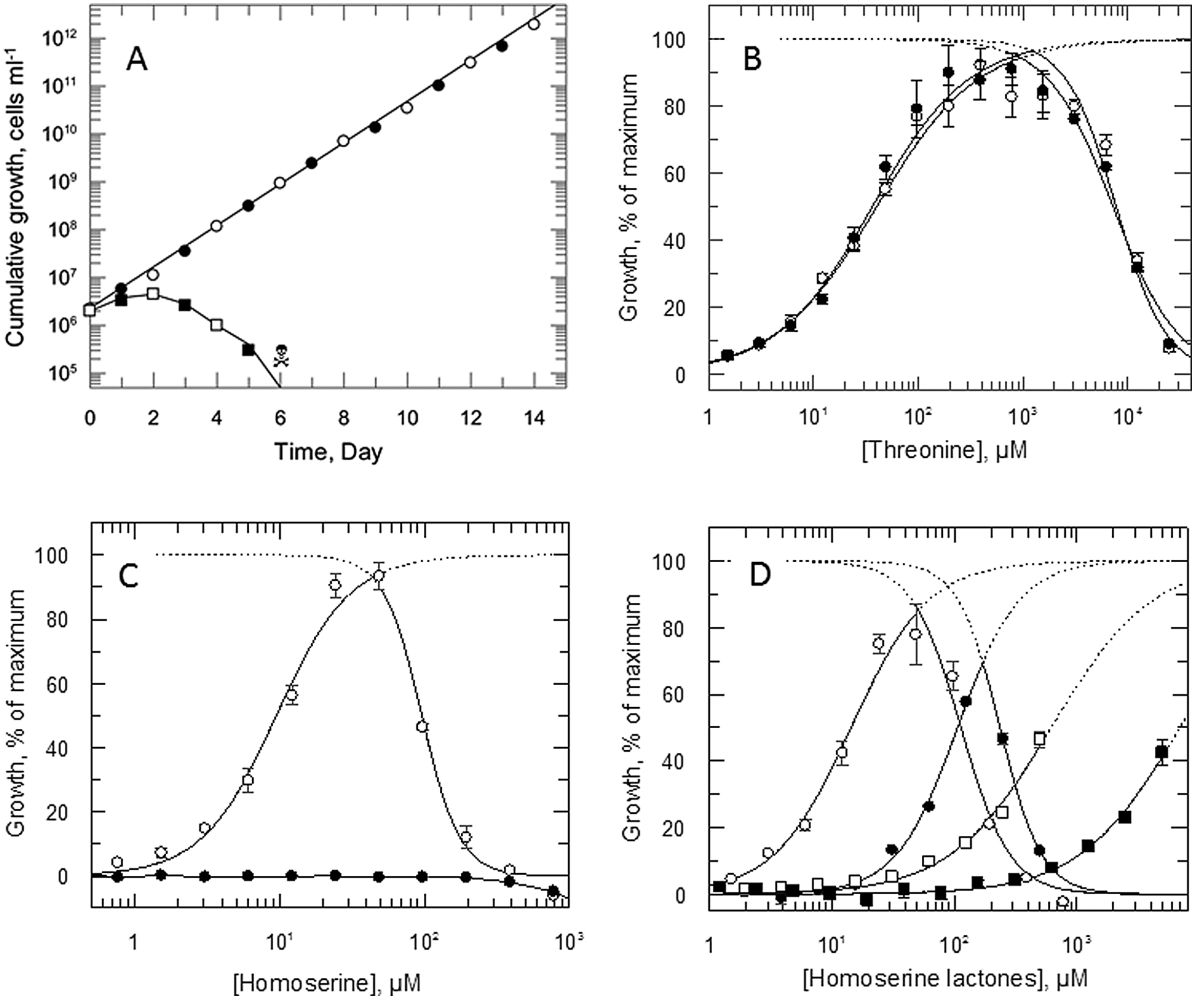
Growth analyses of procyclic WT and DKO cells *in vitro*. A. The cumulative growth curves of WT (open circles) and DKO (closed circles) cells cultured in DTM^TD^ supplemented with 400-μM threonine were determined over 2 weeks. WT (open squares) and DKO (closed squares) cells perished on day 6 in the absence of L-threonine (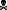). B. Growth of WT (open circles) and DKO (closed circles) cells in varying concentrations of threonine. C. Growth of WT (open circles) and DKO (closed circles) cells in varying concentrations of L-homoserine. D. Growth of WT cells in varying concentrations of L-homoserine lactone (open circles); *N*-3-oxododecanoyl-L-homoserine lactone (closed circles); *N*-3-oxodecanoyl-L-homoserine lactone (open squares); and *N*-3-oxohexanoyl-L-homoserine lactone (closed squares). DKO cells failed to grow with any of these supplements.

The absence of any phenotype in bloodstream forms prompted us to search for a function in the insect stage (procyclic forms) of the *T. brucei* life cycle. WT and DKO bloodstream forms were differentiated to their respective procyclic forms using an established protocol, as described in the methods. Both cell types grew sluggishly for an initial 30 days (doubling times ranging from 33 to 110 h) before rapidly growing cultures were obtained. Subsequent growth analyses were carried out on these established lines. After the initial 30 day adaptation, both WT and DKO parasites continued to grow robustly when sub-cultured in DTM^TD^ plus 400 μM threonine with doubling times of 16.8 ± 0.1 h (Fig. 6A). Both cell types were unable to grow without threonine supplementation, with cell death commencing after 3 days. WT and DKO procyclics had similar requirements for L-threonine (Fig. 6B), with GC_50_ values about five times higher than the bloodstream forms (Table 1). Non-physiological concentrations of threonine (> 1 mM) inhibited growth of both cell lines. However, in marked contrast to bloodstream forms, WT procyclics were able to utilise L-homoserine in place of threonine for growth, while DKO parasites were unable to do so (Fig. 6C). Maximal growth was obtained for WT procyclics at 50 μM homoserine, with higher concentrations inhibiting growth. While having a marginal (∼ fourfold) preference for L-homoserine over L-threonine, WT procyclics were also ∼ 70-fold more susceptible to growth inhibition by L-homoserine versus L-threonine based upon GC_50_ and EC_50_ values (Table 1). These results establish a role for HSK in the insect stage of the *T. brucei* life cycle.

**Table 1:** Comparison of ability of different amino acids and homoserine lactones to support and inhibit growth in procyclic form *Trypanosoma brucei*

	GC_50_ (μM)		EC_50_ (μM)	
	WT	DKO	WT	DKO
L-threonine	36.5 ± 1.9	40.1 ± 5.0	7760 ± 740	7200 ± 520
L-homoserine	9.6 ± 0.58	> 50 000^a^	96 ± 7.3	–
L-homoserine lactone	14.4 ± 0.9	> 25 000^a^	113 ± 8	–
*N*-3-oxododecanoyl-L-homoserine lactone	107 ± 2	> 500^a^	237 ± 5	–
*N*-3-oxodecanoyl-L-homoserine lactone	619 ± 40	> 500^a^	–	–
*N*-3-oxooctanoyl-L-homoserine lactone	> 1250^b^	> 1250^a^	–	–
*N*-3-oxohexanoyl-L-homoserine lactone	6970 ± 520	> 5000^a^	–	–
*N*-3-butyryl-D,L-homoserine lactone	7100 ± 300	> 25 000^a^	–	–

Results are means ± SEM of triplicate measurements.

a No growth observed at maximum concentration tested.

b Fifteen per cent growth observed at solubility limit.

Because DTM^TD^ contains only 100 μM L-aspartate, we tested whether L-aspartate availability was rate limiting for homoserine production. However, WT procyclics were unable to grow in the absence of either L-threonine or L-homoserine, even in the presence of 1 mM L-aspartate. In addition, [^3^H]-aspartate (100 μCi ml^−1^; 8.3 μM) was neither taken up by *T. brucei* nor incorporated into macromolecules when incubated in an aspartate and threonine-deficient DTM (DTM^ATD^). As such, it is doubtful that *T. brucei* can synthesise homoserine *de novo*.

**Figure 7:**
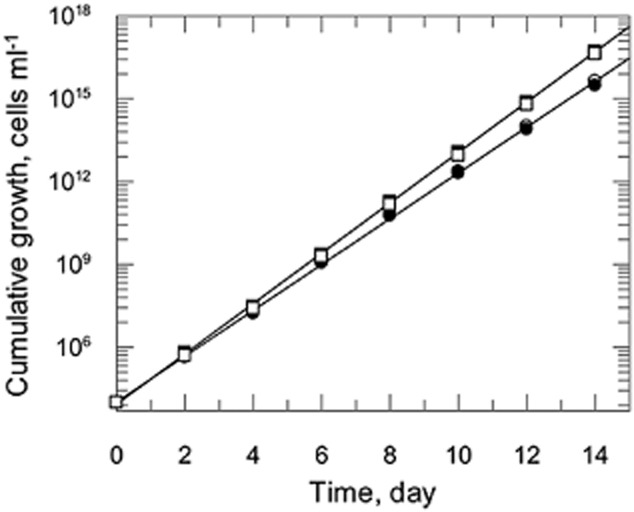
Growth of *Leishmania donovani* promastigotes cultured in medium lacking aspartate and threonine. Cumulative growth curves were determined over 14 days with subculture into fresh medium every 2 days. No additions (open circles); plus 100 μM L-aspartate (closed circles); plus 400 μM L-threonine (closed squares); plus 100 μM L-aspartate and 400 μM L-threonine (open squares).

In stark contrast, *Leishmania donovani* promastigotes were able to grow in DTM^ATD^ without any threonine or aspartate supplementation (Fig. 7). Growth was unaffected by the addition of L-aspartate (100 μM), while doubling time was marginally, but significantly (*P* < 0.0001), reduced from 8.66 ± 0.07 to 7.92 ± 0.05 h when L-threonine (400 μM) was added. Moreover, when incubated with [^3^H]-aspartate in DTM^ATD^ as above, [^3^H]-label was readily detected in both supernatant and pellet extracts of *L. donovani*. Thus, *L. donovani* appears to have a fully functional β-aspartyl phosphate pathway as reported for *L. mexicana* (Saunders *et al*., 2011).

These findings raise a key question: what are the potentially salvageable sources of homoserine in tsetse flies? Male and female tsetse feed exclusively on blood (Jordan, 1993), and females are viviparous, raising a single larva *in utero* which is fed by a milk secretion rich in protein and lipids (Cmelik *et al*., 1969). Mammalian blood does not contain homoserine under normal physiological conditions, so this cannot be the source (Gazarian *et al*., 2002). Moreover, the tsetse genome lacks any genes involved in the threonine *de novo* biosynthesis (International Glossina Genome Initiative, 2014), so the fly itself cannot be the source either. However, tsetse microbiota includes two enteric *Gammaproteobacteria*, the obligate mutualist *Wigglesworthia glossinidia* and the commensal *Sodalis glossinidius*. An analysis of the genome of *S. glossinidius* suggests that this bacterium is capable of synthesising all amino acids, except for alanine (Toh *et al*., 2006), whereas *W. glossinidia* has a reduced capacity (Akman *et al*., 2002). BLAST searches using the threonine biosynthetic pathway enzymes from *E. coli* identified credible candidates (66–80% identity) for all enzymes in the pathway in the *S. glossinidius* genome, but not in *W. glossinidia*. The latter lacks HSK and ThrS, but retains the rest of the pathway for peptidoglycan biosynthesis. The first enzyme in the pathway is a bifunctional aspartokinase-homoserine dehydrogenase, so should also synthesise homoserine. Whether these bacteria can excrete homoserine is not known. A second potential source of homoserine could be acyl homoserine lactones (AHLs), which are secreted by many bacteria for intra- and interspecies communication (Waters and Bassler, 2005). For example, the quorum sensing system in *S. glossinidius* uses *N*-(3-oxohexanoyl) homoserine lactone (3OC_6_HSL) to modulate gene expression in response to bacterial cell density and to oxidative stress (Pontes *et al*., 2008). Accordingly, several commercially available AHLs were tested for their ability to support growth of *T. brucei* in the absence of threonine (Fig. 6D, Table 1). Homoserine lactone and all five AHLs supported growth of WT procyclics to varying degrees, with homoserine lactone > *N*-3-oxododecanoyl-L-homoserine lactone (3OC_12_HSL) > *N*-3-oxodecanoyl-L-homoserine lactone (3OC_10_HSL) > *N*-3-oxohexanoyl-L-homoserine lactone (3OC_6_HSL) > *N*-3-butyryl-DL-homoserine lactone (3C_4_HSL). In contrast, DKO parasites were completely unable to utilise any of these homoserine lactones for growth.

## Discussion

Our study highlights a potentially important role for HSK in the insect stage of the *T. brucei* life cycle under conditions of threonine starvation. Threonine is an important source of acetyl CoA in procyclic *T. brucei*, yet most threonine is metabolised and excreted as glycine and acetate to no apparent purpose, except perhaps for the energy-generating potential of the oxidation step (Cross *et al*., 1975). These authors noted that L-threonine was the sole amino acid to be severely depleted in spent procyclic culture medium. Our amino acid analyses of spent medium confirmed this: after 72 h of culture, we were unable to detect any threonine in our medium. Subsequent studies by others have shown that threonine is indeed the preferred source of acetate for incorporation into fatty acids and sterols, and that ATP is generated from acetyl CoA via the concerted action of the mitochondrial acetate : succinate CoA transferase/succinyl CoA synthetase cycle, as well as from NADH (Millerioux *et al*., 2013).

To confirm the existence of the pathway from homoserine to threonine, we have demonstrated that Tb927.6.4430 encodes a protein with homoserine kinase activity, with properties similar to homoserine kinases from other organisms (Theze *et al*., 1974; Lee and Leustek, 1999; De Pascale *et al*., 2011). The *T. brucei* enzyme behaves more like *A. thaliana* HSK, and, unlike HSK from *E. coli* and *S. cerevisiae*, is not inhibited by L-threonine or L-homoserine (Theze *et al*., 1974; Ramos *et al*., 1991; Lee and Leustek, 1999). Although HSK is expressed in both stages of the *T. brucei* life cycle (Urbaniak *et al*., 2012), nutritional and gene deletion experiments reveal that HSK is not essential for survival or virulence in the clinically relevant mammalian stage, and therefore is not a drug target, unlike in certain fungal pathogens (Kingsbury and McCusker, 2008; 2010a,b[Bibr b25],[Bibr b26],[Bibr b27]).

In contrast to the bloodstream form, HSK does have a potential role to play under conditions of threonine starvation in procyclic form *T. brucei*. Homoserine, homoserine lactone and AHLs were all able to support growth in WT trypanosomes to varying degrees. The fact that none of these metabolites could support growth in HSK null mutants suggests that AHLs must first be degraded to L-homoserine prior to conversion to threonine. The possible carboxypeptidases, acylases or lactonases catalysing this conversion are unknown.

Precise information on the availability of threonine or homoserine in the midgut of the tsetse fly, where the procyclic stage resides, is not available. Tsetse flies are obligate blood feeders and undergo cyclical periods of starvation and feeding every 2–5 days –‘the hunger cycle’. Homoserine is not present in blood, so the initial major source of dietary threonine is from haemoglobin or plasma. The haemoglobin concentration in adult human blood is ∼ 2.5 mM. The protein is an α_2_β_2_ tetramer with nine and seven threonine residues per α and β monomers respectively. Thus, haemoglobin contributes ∼ 80 mM threonine to the blood meal, with threonine in plasma making a minor contribution (0.2–0.7 mM). A similar calculation for proline, a major source of energy for procyclic trypanosomes, yields 70 mM (haemoglobin) and ∼ 0.2 mM (plasma). However, though these sources appear considerable, it should be noted that digestion of the blood meal is complete by 72h and thus trypanosomes are likely to be under nutritional stress thereafter.

Comparative genomics suggests a remarkable metabolic interdependence between the tsetse fly and its microbiota, *S. glossinidius* and *W. glossinidia*. (Some populations also harbour *Wolbachia* which is not considered further in this context as it is localised exclusively intracellularly in germline tissues and not the insect gut.)

**Figure 8:**
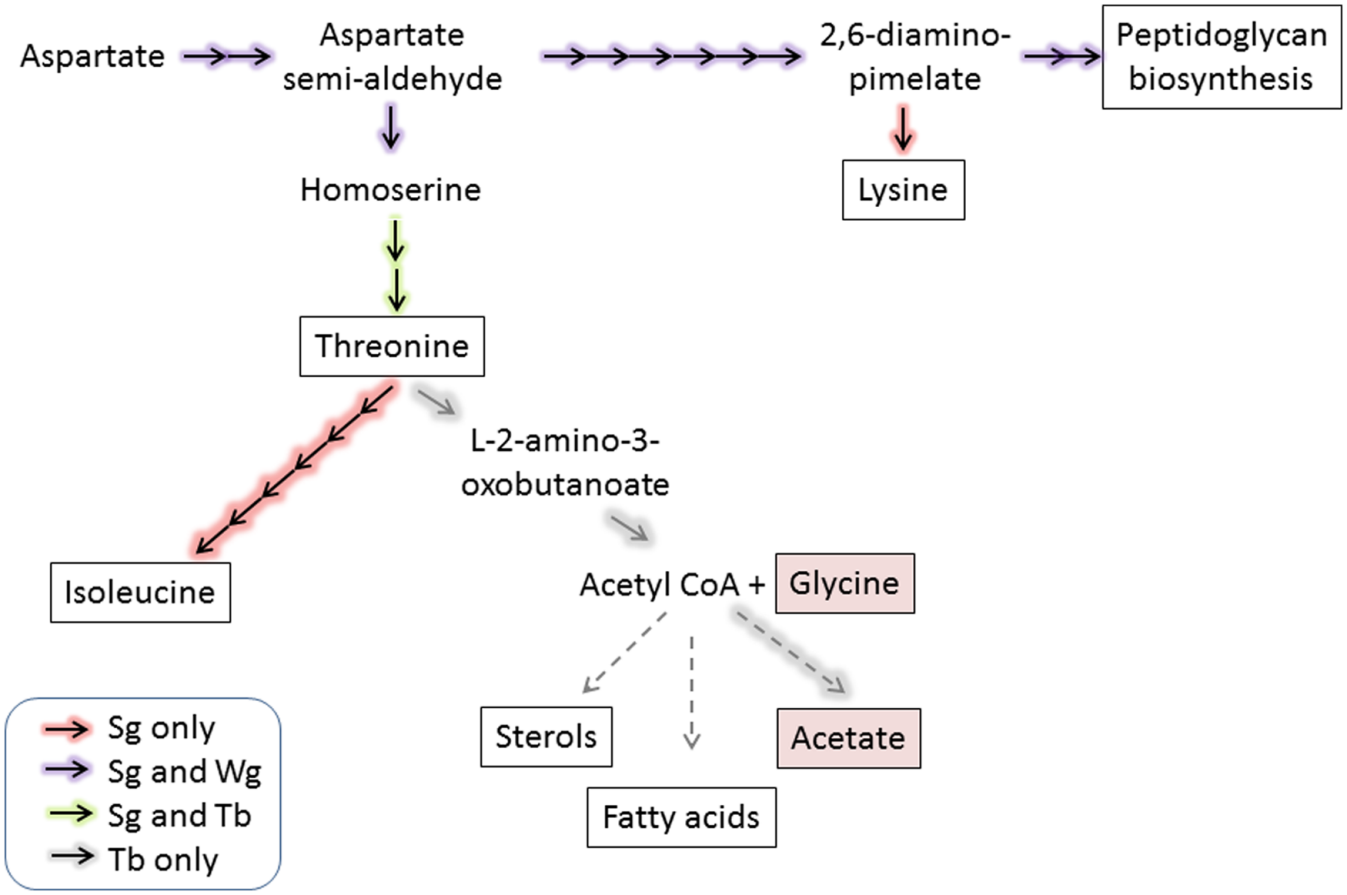
Enzymes predicted by bioinformatic analysis to be present in *Sodalis glossinidius*, *Wigglesworthia glossinidia* and *Trypanosoma brucei*. Key end-products of metabolism are boxed and excreted metabolites boxed and shaded. See legend for further details.

In the case of the obligate symbiont, *W. glossinidia*, despite extensive diminution in its metabolic capabilities, this bacterium has retained the capacity to synthesise cofactors and vitamins (Akman *et al*., 2002). Female flies lacking *W. glossinidia* are sterile, but fertility can be partially restored by supplementing artificial blood meals with B-complex vitamins, suggesting that these vitamins are supplied by *Wigglesworthia*. *W. glossinidia* reside intracellularly in host epithelial cells (bacteriocytes), which form an organ, the bacteriome, in the anterior mid-gut (Wang *et al*., 2013). *W. glossinidia* has retained the ability to convert aspartate to diaminopimelate for the synthesis of peptidoglycan, possibly for protection from the host environment during transmission via the milk secretion to the intrauterine larva. Although predicted to be no longer capable of synthesising threonine, retention of the bifunctional aspartokinase-homoserine dehydrogenase suggests that homoserine may be present as an unwanted intermediate of peptidoglycan biosynthesis (Fig. 8). However, evidence is lacking on whether the homoserine dehydrogenase domain of this protein is still enzymatically active. Moreover, cultivation of *W. glossinidia in vitro* has proved impossible to date, so little is known about its metabolic end-products.

The mutualist endosymbiont, *S. glossinidius*, is widely distributed in tsetse tissues, including the midgut and milk secretions. In contrast to *W. glossinidia*, *S. glossinidius* has retained the ability to synthesise diaminopimelate, as well as threonine (Fig. 8), in addition to all other protein amino acids, except alanine (Toh *et al*., 2006). Interestingly, *S. glossinidius* is also predicted to possess a putative threonine export protein (Toh *et al*., 2006). This appears similar to the situation in *E. coli* (RhtA, RhtB and RhtC) and *Corynebacterium glutamicum* (ThrE) used for the commercial production of this essential amino acid (Dong *et al*., 2011). Of these permeases, only RhtA is reported to efflux both L-threonine and L-homoserine (Dong *et al*., 2011). However, the putative export function and amino acid specificity of the *S. glossinidius* permease has not been studied. Another potential source of homoserine is the quorum-sensing AHL *N*-3-oxohexanoyl homoserine lactone secreted by *Sodalis* (Pontes *et al*., 2008). Unfortunately, the concentration of this AHL was not reported by the authors, so it is difficult to assess the potential nutritional role of this secondary metabolite. This would involve knowledge of the bacterial load, the rate of synthesis, diffusion and degradation of AHL in the tsetse. Very few publications report the concentrations of AHLs in other bacterial systems and those that do vary widely: ∼ 0.1 μM for *Vibrio fischeri* in symbiotic squid light organs (Boettcher and Ruby, 1995), 16 μM in stationary phase *Agrobacterium tumefaciens* (Zhu *et al*., 1998) and > 600 μM in *Pseudomonas aeruginosa* biofilms (Charlton *et al*., 2000). However, indirect evidence can be drawn from studies on *S. glossinidius*-free flies obtained by treatment with streptozotocin, an antibiotic that had no effect on *Wigglesworthia*. These flies showed a deceased longevity, with no loss in fertility, but became significantly more resistant to trypanosome infections (Dale and Welburn, 2001). Although the authors attributed this effect to the bacterium's chitinolytic activity, it is also possible that this could be attributed to loss of a source of homoserine.

In conclusion, our findings indicate that L-homoserine or AHLs can substitute for the essential amino acid L-threonine in procyclic forms of *T. brucei*. Although biochemical evidence for the source of these precursors is lacking, bioinformatic analysis of the tsetse fly endosymbionts suggests a number of plausible sources. Our hypothesis offers an explanation for the trypanosome retaining a partial pathway, in contrast to the leishmania parasite that retains the complete pathway. Further work is needed to test which of these hypotheses is correct.

## Experimental procedures

### Organisms and reagents

Chemicals and reagents used in this study were of the highest grade and purity available. L-homoserine, L-homoserine lactone dihydrochloride and all AHLs were purchased from Sigma Aldrich. *T. brucei* bloodstream form ‘single marker’ S427 (*T7RPOL TETR NEO*) was cultured at 37°C in HMI9T (Greig *et al*., 2009), supplemented with 2.5 μg ml^−1^ G418 (Geneticin, Invitrogen). *L. donovani* promastigote cell line LdBOB (derived from MHOM/SD/62/1S-CL2D) was grown in M199 plus supplements (Goyard *et al*., 2003) at 28°C. Threonine-deficient trypanosome base media (TBM^TD^) was prepared as described for TBM (Ong *et al*., 2013), with threonine excluded from the Iscove's modified Dulbecco's MEM component and supplemented with 10% dialysed bovine foetal calf serum (PAA Laboratories). Threonine-deficient differentiation medium (DTM^TD^) is based on DTM (Ziegelbauer *et al*., 1990) and was prepared by excluding threonine from the MEM essential amino acids solution formulation and replacing normal FCS with 15% dialysed bovine FCS. Cell densities were determined using the CASY TT cell counter (Schärfe).

### Generation of protein expression and knockout constructs

PCR primers were designed using the *T. brucei HSK* sequence in GeneDB (Tb927.6.4430) as a template to generate constructs for protein expression and genetic manipulation (Table S1). The *HSK* open reading frame (ORF) was PCR-amplified from *T. brucei* genomic DNA using ORF/XhoI_s and ORF/BamHI_as primers (Table S1) and *Pfu* polymerase. The resulting ∼ 1 kb PCR product was cloned into the Zero Blunt®TOPO shuttle vector (Life Technologies), before subcloning into the *E. coli* expression vector pET15b modified to include a tobacco etch virus protease cleavage site for recombinant protein expression. The 5′- and 3′-untranslated regions (UTRs) of *HSK* were similarly PCR-amplified from *T. brucei* genomic DNA using gene knockout primers (5′UTR/NotI_s, 5′UTR/HindIII_as, 3′UTR/BamHI_s and 3′UTR/NotI_as; Table S1) and *Pfu* polymerase. The amplified regions were used to assemble the replacement cassettes containing the selectable drug resistance genes puromycin *N*-acetyl transferase (*PAC*) and hygromycin phosphotransferase (*HYG*), exactly as previously described (Martin and Smith, 2005). All constructs were confirmed by DNA sequencing (www.dnaseq.co.uk).

### Recombinant expression and purification of *Tb*HSK

Recombinant *Tb*HSK was expressed in *E. coli* strain ArcticExpress (DE3) RP (Agilent Technologies). Transformed cells were cultured in autoinduction medium plus 100 μg ml^−1^ ampicillin at 37°C with shaking at 200 r.p.m. until OD 0.8–1.0 was achieved. Cultures were further incubated for 72 h at 12.5°C, with shaking at 200 r.p.m. before cells were harvested by centrifugation (3000 *g*, 20 min, 4°C). Cells were resuspended in lysis buffer (50 mM Tris-HCl and 200 mM NaCl, pH 7.5) supplemented with ethylenediaminetetraacetic-acid-free complete protease inhibitor cocktail (Roche) and lysed using a continuous cell disruptor (Constant Systems) at 30 000 Psi. Lysates were clarified by centrifugation (40 000 *g*, 30 min, 4°C) and recombinant proteins purified using nickel affinity chromatography. Supernatants were applied to a 5 ml Ni^2+^ column (GE Healthcare), pre-equilibrated in lysis buffer and connected to an AKTA™ FPLC purifier. Bound proteins were eluted using a gradient of 0–100% 1M imidazole in lysis buffer and analysed by SDS-PAGE using a NuPAGE Novex 4–12% Bis-Tris gel (Life Technologies). Proteins were visualised and purity assessed by Coomassie Brilliant Blue staining.

### Enzyme kinetics

The activity of recombinant *Tb*HSK was determined using a previously described spectrophotometric assay (Lee and Leustek, 1999) on a UV-1601 spectrophotometer (Shimadzu). *Tb*HSK was pre-equilibrated with 0.25 mM NADH, 1.2 mM phosphoenolpyruvate, 3 mM ATP, 10 mM MgSO_4_ and the coupling enzymes [pyruvate kinase (10 U) and lactate dehydrogenase (15 U), Sigma Aldrich] for 1 min at 25°C. Enzymatic reactions were initiated by the addition of L-homoserine. Initial rates of NADH oxidation was measured for 120 s at 340 nm and converted to molar units using the extinction coefficient 6.22 mM cm^−1^. One unit of enzyme activity is defined as one micromole of substrate used per minute. Except for pH optimum determination, all enzymatic assays were carried out in 50 mM HEPES buffer, adjusted to pH 7.4 and an ionic strength of 100 mM using KOH and KCl. The pH optimum of *Tb*HSK (0.7 μM) for L-homoserine (3000 μM) was determined using constant ionic strength overlapping buffers as previously described (Ong *et al*., 2011). The *K*_m_^app^ of *Tb*HSK for L-homoserine was determined by measuring the activities of a fixed enzyme concentration (0.7 μM) in the presence of varying concentrations of L-homoserine (23–3000 μM). The results were analysed using GraFit and fitted to the Michaelis–Menten equation.

### Generation of *T. brucei* transgenic mutants

Knockout plasmids were linearised using NotI, precipitated with ethanol and resuspended in sterile water (1 μg ml^−1^). Wild-type *T. brucei* (4 × 10^7^ cells) were harvested and resuspended in reagents from the Human T cell Nucleofector kit, as per manufacturers’ instructions. Linearised DNA (5 μg) were added and cells were electroporated using programme X-001 of the Nucleofector II electroporator (Amaxa) (Burkard *et al*., 2007). A single knockout cell line of *HSK* (SKO^HYG^) was first generated by replacing the first allele with the *HYG* gene. SKO^HYG^ parasites were selected by culturing in the continuous presence of hygromycin (4 μg ml^−1^). SKO^PAC^ parasites were prepared in a similar fashion using 0.1 μg ml^−1^ puromycin for selection. The remaining *HSK* allele in an established SKO^HYG^ clone was subsequently replaced by transfection with the *PAC* construct and selection with hygromycin and puromycin in order to generate a DKO cell line. DKO parasites were selected by culturing in the continuous presence of both hygromycin and puromycin. Transfected parasites were cloned by limiting dilution at each respective stage.

### Southern blot analysis

Digoxigenin-labeled 3′-UTR of *T. brucei HSK* was generated by PCR amplification (with primers previously described for generating knockout constructs) using the PCR DIG Probe Synthesis Kit (Roche) as per manufacturer's instructions. Using the resulting product as a probe, Southern analysis of genomic DNA (5 μg) samples digested with the restriction endonuclease AatII was carried out exactly as previously described (Wyllie *et al*., 2009).

### *In vivo* studies

All animal experiments were subject to local ethical review and performed under the Animals (Scientific Procedures) Act 1986 in accordance with the European Communities Council Directive (86/609/EEC). Groups of five mice were infected by intraperitoneal injection (10^4^ parasites), with bloodstream-form *T. brucei* cultured in HMI9T. Infections and parasitaemia were monitored as previously described (Sienkiewicz *et al*., 2008), with animals reaching a terminal parasitaemia (> 10^8^ cells ml^−1^) euthanized and recorded as dead on the same day.

### Differentiation of bloodstream *T. brucei* to procyclic form

Differentiation of bloodstream *T. brucei* to procyclic form was carried out as previously described (Ziegelbauer *et al*., 1990), with minor modification. Bloodstream form *T. brucei* cultured in HMI9T were harvested, washed once and resuspended (3 × 10^6^ cells ml^−1^) in DTM^TD^ plus 400 μM threonine and CCA (3 mM sodium citrate + 3 mM sodium cis-aconitate). Cultures were incubated at 28°C with cell densities maintained between 1 × 10^6^ and 8 × 10^6^ ml^−1^. Rapidly growing differentiated cells were established in DTM^TD^ plus 400 μM threonine after 30 days (CCA excluded after day 7) before further analyses.

### Ability of *T. brucei* to utilise threonine, homoserine and homoserine lactones for growth

The ability of threonine, homoserine and homoserine lactones to support the growth of bloodstream and procyclic forms of *T. brucei* were investigated in TBM^TD^ and DTM^TD^ respectively. Concentrations required to support growth were determined in 96-well microtitre plates in a final culture volume of 200 μl per well and an initial parasite seeding density of 2.5  ×  10^3^ cells ml^−1^ (bloodstream) or 5  ×  10^5^ cells ml^−1^ (procyclics). Stock solutions of threonine, L-homoserine, L-homoserine lactone and *N*-butyryl-D,L-homoserine lactone were prepared in water, while other AHLs were dissolved in dimethylsulfoxide. Appropriate amounts of solvent were added in control samples. Cultures were incubated for 48 h (procyclic form) or 72 h (bloodstream form) before cell densities were determined using a resazurin-based assay (Jones *et al*., 2010). Growth is expressed as a percentage of maximum growth relative to the same cell line grown in the presence of 400 μM (procyclic form) or 800 μM L-threonine (bloodstream form). These threonine concentrations correspond to the original levels present in DTM and TBM. The concentration of supplement required to support 50% maximum cell growth (GC_50_) was determined by plotting cell density versus amino acid concentration and analysed by 2-parameter non-linear regression using GraFit.

### Radiolabelling studies

*Trypanosoma brucei* procyclics *and L. donovani* promastigotes were harvested by centrifugation (800 *g*, 10 min, 4°C) and resuspended (1 × 10^7^ cells ml^−1^) in fresh DTM^ATD^ or transport buffer (Ali *et al*., 2013) containing-L- [2,3-^3^H]-aspartate (Perkin Elmer, final concentration 8.3 μM, 100 μCi ml^−1^). Cells were harvested by centrifugation after 1, 2, 4, 24 h and washed twice with transport buffer. The resulting cell pellets were heat treated with dabsyl chloride to derivatise the amino acids as previously described (Sethuraman *et al*., 2004). Samples were centrifuged (16 000 *g*, 10 min, 22°C) and 0.02 ml of the resulting supernatants was added to 1 ml of scintillation fluid. Pellets were solubilised in 0.1 ml 0.1 M NaOH and mixed with 1 ml scintillation fluid. Radioactivity was then determined using a Beckman scintillation counter.
