# Supplementary material for: Homoserine and quorum-sensing acyl homoserine lactones as alternative sources of threonine: a potential role for homoserine kinase in insect-stage Trypanosoma brucei
Source: Mol Microbiol. 2014 Nov 25;95(1):143–56. doi: 10.1111/mmi.12853 (PMC4460637; doi:10.1111/mmi.12853)
Supplement: Supplementary file 1 [file mmi0095-0143-sd1.pdf]

Supplementary Material

Table S1 Cloning primers

Upper case letters refer to nucleotides corresponding to gene sequences in *T. brucei*; lower case refers to additional sequences used in generating constructs. Restriction endonuclease sites are underlined.

Primer name	Primer sequence
ORF/XhoI_s	5´ctcgagATGTGTGTACTTCCTAGGAAG 3´
ORF/BamHI_as	5´ggatccCTAAGTCGATACATAAACAAGATTAT 3´
5´UTR/NotI _s	5´ataagaatgcggccgcCGACTATCCTCTTTCGTCGAA 3´
5´UTR/HindIII_PmeI_as	5´gtttaaacttacggaccgtcaagcttCCGCTAAAATCACCCAGTTAG 3´
3´UTR/PmeI_BamHI_s	5´gacggtccgtaagtttaaacggatccTTATTTCTTTTGTCCTTTGGT 3´
3´UTR/NotI_as	5´ataagtaagcggccgcGATATGGAAATGCTAGAACTT 3´
